# Speech Emotion Recognition with Heterogeneous Feature Unification of Deep Neural Network

**DOI:** 10.3390/s19122730

**Published:** 2019-06-18

**Authors:** Wei Jiang, Zheng Wang, Jesse S. Jin, Xianfeng Han, Chunguang Li

**Affiliations:** 1 College of Intelligence and Computing, Tianjin University, Tianjin 300072, China; jiangweitju@163.com (W.J.); jinsheng@tju.edu.cn (J.S.J.); hanxianf@163.com (X.H.); 2 School of Computer Information and Engineering, Changzhou Institute of Technology, Changzhou 213032, China; licg@czu.cn

**Keywords:** human–computer interaction (HCI), speech emotion recognition, deep neural architecture, heterogeneous feature unification, fusion network

## Abstract

Automatic speech emotion recognition is a challenging task due to the gap between acoustic features and human emotions, which rely strongly on the discriminative acoustic features extracted for a given recognition task. We propose a novel deep neural architecture to extract the informative feature representations from the heterogeneous acoustic feature groups which may contain redundant and unrelated information leading to low emotion recognition performance in this work. After obtaining the informative features, a fusion network is trained to jointly learn the discriminative acoustic feature representation and a Support Vector Machine (SVM) is used as the final classifier for recognition task. Experimental results on the IEMOCAP dataset demonstrate that the proposed architecture improved the recognition performance, achieving accuracy of 64% compared to existing state-of-the-art approaches.

## 1. Introduction

As an expression of emotion, sound plays a very important role in human communication, and it has drawn wide attention from many institutions in the research fields of human–computer interaction (HCI) or human–robot interaction [1,2]. For instance, in the context of human–robot interaction, if the robot is able to recognize a person’s emotion through conversation, it could adopt appropriate behavior to interact well with that speaker.

The commonly used emotional representation methods are mainly divided into two types, namely the discrete emotion description model and the continuous emotion description model [3,4,5]. The former describes the human emotional states such as happy, sad, disgust, fear, surprise, anger, neutral, etc., in people’s daily life. The latter is also called dimensional emotional model and uses a continuous emotional space to describe the emotion. The commonly used dimension emotion model is a two-dimensional arousal-valence model [6,7]. In this work, the discrete emotion description model is adopted to perform the human emotion recognition.

As for the emotion analysis and detection, discriminative features extraction from speech data is one of the key factors in the success of a recognition model. The single feature vector extracted from one aspect finds it difficult to meet the demand due to the emotional gap between human emotions and single feature for emotion recognition. In the large amount of research on speech emotion recognition, there are many widely used low-level handcrafted features for sentiment analysis and detection in speech signal processing [8,9,10]. Nevertheless, with the advent of deep neural networks (DNNs), which have been proven to have the capabilities of extracting better feature representation, high-level features were extracted from speech data and employed for recognition task in numerous researches [11,12,13]. After obtaining a variety of acoustic feature data, those feature information should be fully used for improving emotion recognition performance. Some studies [10,11] demonstrated that integration strategy is effective in emotional classification. However, in essence, these different types of feature representations are generally heterogeneous, so a basic challenge is how to effectively integrate this heterogeneous information for better recognition performance.

Another challenge in speech emotion classification is the fusion of the multiple features. A number of previous researches [14,15,16,17,18,19,20] have been reported which focused on major fusion strategies. While most of the above mentioned fusion methods yielded good performance, they almost simply concatenated the multiple features into a single high-dimensional feature vector and fed it into a final classifier or a shallow fusion model which has difficulty in joining learning intrinsic correlations between different acoustic feature representations. Thus a suitable fusion structure with deep learning network is required to learn discriminative features and discover high-level associations from multiple acoustic features for emotion prediction.

To address the problems mentioned above, this paper explores how to make full use of the low-level and high-level acoustic features obtained from different aspects, and how to take full advantage of the DNNs’ ability to fuse the multiple information for achieving better classification performance. In comparison with the existing researches on speech emotion recognition and classification, our key contributions are as highlighted below and are detailed in the next section.

- Different from directly using varieties of acoustic features such as handcrafted features or high-level features for emotion recognition, we propose a hybrid framework which could discover the informative feature representations effectively from the heterogeneous acoustic feature groups to eliminate the redundant and unrelated information.
- After investigating different types of fusion strategy, a fusion network module based on deep neural networks is proposed to fuse the informative feature representations for better results of speech emotion prediction.
- We compare the proposed framework with other prominent methods for acoustic emotion recognition. Extensive experimental results on the emotional dataset show that our framework achieves promising performance which demonstrates the effectiveness of our approach.

The remainder of this paper is organized as follows. Some important and related work on speech emotion recognition are reviewed in Section 2. In Section 3, we present the proposed hybrid architecture for speech emotion classification and describe it in detail. Section 4 represents the experimental results and analysis. The conclusions and future work are given in Section 5.

## 2. Related Work

In a speech emotion recognition system, features extraction, features unification and fusion network are the most important processes for better performance. Therefore, in the following parts, we review some important work related to the above mentioned processes on account of some pivotal concepts and techniques.

### 2.1. Acoustic Features Extraction

The large number of studies for speech emotion recognition have focused on extracting speech features as different emotional representations [8,9,10,11,12,13,21,22,23,24]. In recent years, the acoustic features widely used for emotion analysis and recognition can be categorized into two classes, low-level features and high-level features.

Generally, the low-level acoustic features, including prosodic (such as fundamental frequency, speech rate, intensity, duration, energy, pitch, etc.), voice quality (such as format frequency and bandwidth, jitter and shimmer, glottal parameter, etc.) [25], spectral (such as spectrum cut-off frequency, spectrum centroid, correlation density and mel-frequency energy, etc.), cepstral (such as Mel-Frequency Cepstral Coefficients (MFCCs), Linear Prediction Cepstral Coefficients (LPCC), etc.), and so on. Voice quality features are extracted within a 40 ms frame with a window shift of 10 ms, and cepstral-based features are extracted within a 25 ms frame with a window shift of 10 ms [9].

Nowadays, more and more researchers have focused on deep learning methods because of their superior abilities for automatically learning discriminative high-level representations from speech data [26,27,28,29,30] for emotion recognition. In [29], Lakomkin et al. introduced several models which utilized neural representations inferred by training on large speech databases for emotion recognition task. The experiments on the Interactive Emotional Motion Capture (IEMOCAP) database [31] achieved 58% and outperformed the baseline recurrent neural network. Gu et al. [30] proposed a hierarchical multimodal architecture with attention and word-level fusion to classify emotion on the IEMOCAP dataset. The introduced model achieved the performance with 62% accuracy and outperformed state-of-the-art approaches.

In those researches discussed above, different kinds of acoustic features were extracted for affective analysis and detection. In general, high-level features are extracted by using pre-trained deep neural networks such as SoundNet [27], and low-level handcrafted features are extracted by using open source framework such as OpenSmile [32]. However, most of the methods mentioned above just simply employed the extracted low-level or high-level features for current tasks. They did not make full use of the two kinds of features for better performance and they ignored the intrinsic relationship between the low-level and high-level features.

### 2.2. Heterogeneous Acoustic Features Processing

After obtaining various acoustic features, how to effectively integrate multiple features for better recognition performance becomes a very crucial issue.

Numerous studies have shown that multiple deep neural networks could learn a discriminative representation of the input signal directly from audiovisual data for classification tasks. In [33], Ngiam et al. proposed a series of frameworks to learn features over multiple modalities by combining different deep learning models. Extensively experiments on the Clemson University Audio Visual Experiments (CUAVE) database [34] and Audio-Visual of isolated Letters (AVLetters) dataset [35] demonstrated the best published audiovisual classification and effective shared representation learning. Srivastava et al. [36] proposed a Deep Belief Network (DBN) for learning a generative model of multiple modalities which defined a probability density over the space of multimodal inputs. The experiments demonstrated that model significantly outperformed SVM and Linear Discriminant Analysis (LDA) models on classification and information retrieval tasks.

In addition, some kernel methods, such as multiple kernel learning (MKL), etc., have been frequently utilized as a strategy to take advantage of these multiple features recently. The objective in MKL is to jointly learn a set of kernels and parameters which act as weights for those multiple kernels instead of a single kernel function for better recognition results [37,38]. After a lot of research for similarities and differences between MKL algorithms, Nen et al. [37] classified and reviewed MKL methods in recent years. Extensive experiments on real datasets showed that using MKL strategy instead of a single kernel was useful and could achieve better performance. In [38], Nilufar et al. designed a special MKL method for difference of Gaussians scale selection/weighting and handled high dimensional scale-space data. They performed extensive experiments on several datasets showed the framework combined MKL with special strategy yielded encouraging results against other methods.

Nevertheless, these methods mentioned above just made use of the multiple acoustic features to achieve better result by deploying deep neural networks or MKL. In those studies, they ignored the problem that these features were essentially heterogeneous, because the variety of features were extracted according to different aspects of the original task, especially some of them were still low-level features. Therefore, an effective approach should be needed to deal with the obstacle of unifying the heterogeneous feature representation for improving performance.

### 2.3. Multiple Acoustic Features Fusion

In order to achieve better performance, a fusion method of combining different feature information is indispensable. In many studies in recent years, varieties of fusion strategies have been adopted to do the speech emotion recognition.

Based on different strategies, the fusion methods can be broadly divided into feature-level fusion (or early fusion), model-level fusion (or middle fusion) and decision-level fusion (or late fusion). Feature-level fusion method simply and directly concatenates multiple types of features into a high-dimensional feature vector. Afterwards, the single feature vector is fed into a classifier or other models for training to get better performance in this way [39,40]. Mansoorizadeh et al. [39] presented an asynchronous feature-level fusion approach which generated an unified hybrid features space for clustering or classification of the multimedia content including speech speech prosody and facial expressions. The experiment on two audiovisual emotion databases showed that the proposed method obtained the significantly higher performance. In [40], Gu et al. proposed a deep multimodal architecture to predict emotional states from speech. They extracted high-level features from text and audio, and directly concatenated two types of features into a single vector to fuse by using a three-layer deep neural network. The proposed method achieved promising performance on the IEMOCAP dataset.

However, simply concatenating the multiple features will bring noise from each single modal into the final feature vectors, which ultimately leads to low accuracy of recognition or classification. Whereas, unlike the feature-level fusion strategy, decision-level fusion method fuses the decisions derived from those features by utilizing a special rule. Specially, in decision-level fusion strategy, each type of feature is independent and modeled with a separate classifier like SVM, logistic regression(LR). In [19], inspired by the powerful feature learning ability of DNN, Zhang et al. proposed a hybrid deep model for audiovisual emotion recognition. Several kinds of DNN models were deployed to extract features from multimodal data. The decision-level fusion strategy was used to obtain the promising performance in the proposed method. Kim et al. [20] proposed a multi-modal emotion recognition by using semi-supervised learning and multiple neural networks. Multi-modal features were extracted from videos through multiple deep learning models. Finally, a decision-level fusion named adaptive fusion was applied to achieve a competitive classification result.

Another fusion strategy, namely model-level fusion, fuses varieties of features which are acquired from multiple models. Typical model-level fusion approaches are concatenating the outputs from hidden layers of different neural networks or other models. In [41], Missaoui et al. proposed a model level fusion approach for combining heterogeneous sets of features for the continuous Hidden Markov Model (HMM) classifier. Extensive experimental results on a large collection of ground penetrating radar (GPR) alarms showed that the model-level fusion achieved the promising performance compared to the baseline HMM when single feature was used independently and when both features were combined with equal weights.

Those researches adopted different kinds of fusion strategies for better performance, however, they overlooked that the multiple features were heterogeneous essentially. Actually, no matter which of the most suitable fusion strategy was used, those approaches in which heterogeneous features were acted as input data would not achieve the best result.

Our work is motivated by some opinions and ideas from the research above mentioned. A hybrid deep neural architecture is proposed to extract distinguished feature representations from heterogeneous acoustic features in this work. In order to achieve better performance, we employ a four-layer deep neural network, which acts as a feature fusion framework, to capture the associations between the unified features. The proposed architecture and methodology in our work are described in detail in next section.

## 3. Proposed Speech Emotion Recognition Architecture

**Figure 1:**
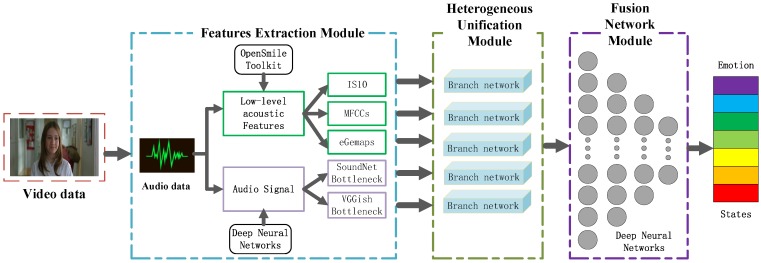
The proposed speech emotion recognition architecture.

In this section, a hybrid speech emotion recognition architecture is proposed, as shown in Figure 1.

The whole emotion recognition architecture consists of three modules, namely, a features extraction module, a heterogeneous unification module and a fusion network module.

To make full use of the low-level and high-level acoustic features, several kinds of features are extracted from the input audio data in the features extraction module of the proposed framework in Figure 1. The three kinds of low-level acoustic features are highlighted in the green boxes and the two high-level acoustic features are highlighted in the lilac boxes. The core of the proposed architecture is the heterogenous unification module which consists of five branch networks. The heterogenous unification module aims to deal with the heterogeneity problem existing among the different features generated from the features extraction module. Each single heterogeneous feature is fed into the branch of multiple deep neural networks in the heterogeneous unification module afterwards. As a result, the heterogeneous features from the heterogeneous space are converted into uniform types in the unified space. Another important module in the proposed architecture is the fusion network module which is a four-layers deep neural network. By taking full advantage of the DNNs ability, the fusion network module concatenates the multiple unified features as a joint feature representation and exploits the associations between them to perform the final recognition task. The three modules are described in detail in the following section.

### 3.1. Features Extraction Module

It can be seen from Figure 1, the first step in features extraction module is to extract audio data from the video data. Mentioned in precious section, the open source toolkit OpenSmile [32] is frequently utilized to extract the low-level statistical acoustic features which are widely used and proved to be excellent in speech emotion recognition tasks. Multiple low-level acoustic features, such as IS10, MFCCs, eGemaps [42], are extracted for speech emotion recognition in this work.

The acoustic feature set IS10 could be extracted by OpenSmile with the confguration in INTERSPEECH 2010 Paralinguistic challenge [43] which includes low-level acoustic features, such as energy, pitch, jitter and so on. MFCC, the most well-known spectral feature, is a popular technology and based on the known variation in the critical frequency bandwidth of the human ear. MFCCs are coefficients that collectively make up an MFC [44] from speech data. They are derived by decorrelating the output logarithmic energies of the filter banks, which consist of triangular filters, linearly spaced on the Mel frequency scale [45]. Like IS10, MFCCs and eGemaps are extracted by OpenSmile with the corresponding configuration files.

Besides, we extract high-level acoustic features from the deep speech recognition networks because of their superior capability for automatically generating informative representations from the audio data. Nevertheless, due to the lack of sufficient samples in the speech emotion datasets, it is hard to obtain the discriminative feature representations from the complex deep learning networks which should be trained well. Therefore, in many researches, bottleneck features extracted from fine-tuned deep neural networks are often proposed for classification tasks.

In this work, besides low-level handcrafted features, high-level acoustic feature presentations named SoundNet [27] bottleneck feature and VGGish [46] bottleneck feature, are considered for speech emotion recognition task. SoundNet can capitalize large amounts of unlabeled sound data collected in the wild to learn rich natural sound representations. In our work, the SoundNet and VGGish network are acted as the high-level feature extractors which have been proved to be highly efficient for audio classification task.

As a result, we can acquire several types of low-level acoustic features by using OpenSmile. While, SoundNet bottleneck feature and VGGish bottleneck feature are obtained from the output of pre-trained neural networks given by the hidden layer.

### 3.2. Heterogeneous Unification Module

Through the features extraction module, various acoustic features are obtained by using a number of feature extraction approaches for emotion classification. Nevertheless, the multiple features are generally high-dimensional and heterogeneous with distinct distributions in a variety of different feature spaces. So, it is difficult to exploit the intrinsic relation between them at the low-level representation spaces and fuse them for good recognition performance.

In the light of the idea [47,48], a heterogeneous unification module is introduced in detail to convert the heterogeneous space of various features into a unified representation space by deploying unsupervised feature learning technique based on deep neural networks. Because of the ability of feature learning in an unsupervised way, the autoencoder structure is constantly adopted to learn a new non-linear transformation at the high-level space from the formerly obtained feature representation space.

In the following sections, autoencoder structure and its variants, which are employed to yield abstract high-level representations in the proposed architecture, are introduced and discussed in detail.

#### 3.2.1. AutoEncoder

**Figure 2:**
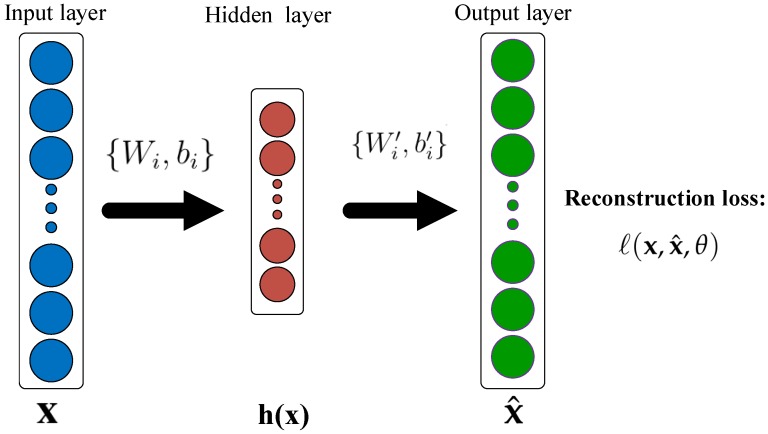
The architecture of an autoencoder.

An autoencoder is a multiple layers feed-forward neural network, which is shown in Figure 2.

Given a training set of examples $\left(x_{i},y_{i}\right)$ of *q* instances, we define: Training data:$\left\{\left(x_{i},y_{i}\right)|x_{i}\in R^{N},y_{i}\in \left\{-1,1\right\}\right\}$, St.i=1,2,3,...,q. Where $x_{i}$ is from the N-dimensional feature space x and $y_{i}$ indicates the class, to which the corresponding $x_{i}$ belongs.

In response to an input $x_{i}\in R^{N}$, the hidden representation $h\left(x_{i}\right)\in R^{M}$ is:

(1) $$h\left(x_{i}\right)=f\left(W_{i}\cdot x_{i}+b_{i}\right).$$

where f(·) is a non-linear transformation function, $W_{i}\in R^{M\times N}$ denotes a weight matrix and $b_{i}$ is a bias vector. Typically, the rectified linear unit is used as the non-linearity after the output of the last layer of the encoder. The network output finally decodes the hidden representation $h(x_{i})$ back into a reconstruction $\hat{x_{i}}\in R^{N}$:

(2) $$\hat{x_{i}}=g\left(W_{i}^{\prime }\cdot h\left(x_{i}\right)+b_{i}^{\prime }\right).$$

where g(·) is a non-linear activation function, $W_{i}^{\prime }\in R^{N\times M}$ denotes a weight matrix and $b_{i}^{\prime }$ is a bias vector.

Thus, the parameters $\left\{W_{i},b_{i}\right\}$ represent the connections from the input to the hidden layers, and the parameters $\left\{W_{i}^{\prime },b_{i}^{\prime }\right\}$ represent the connections from the hidden layers to the output layer. Then, we must specify a training loss function to minimize the reconstruction loss $\ell (x,\hat{x},\theta )$ as either the traditional squared error:

(3) $$\ell \left(x,\hat{x},\theta \right)={∥x-\hat{x}\left(\theta \right)∥}^{2}.$$

As for binary observations, another natural choice is the cross-entropy loss:

(4) $$\ell \left(x,\hat{x},\theta \right)=-\sum_{i=1}^{q}\left[x_{i}\log\hat{x_{i}}\left(\theta \right)+\left(1-x_{i}\right)\log\left(1-\hat{x_{i}}\left(\theta \right)\right)\right].$$

where $\theta =\{W_{i},b_{i},W_{i}^{\prime },b_{i}^{\prime }\}$ is the parameters in Equations (3) and (4).

Training the autoencoder corresponds to optimizing the parameter θ to reduce the reconstruction error $\ell (x,\hat{x},\theta )$ on the training examples, usually with (mini-batch) stochastic gradient descent as in the training of neural networks.

#### 3.2.2. Denoising AutoEncoder

The denoising autoencoder (DAE) [49] is an extension of a basic autoencoder. The main idea behind DAE is to train a basic autoencoder which could reconstruct the input data from a corrupted version that has been artificially added with random noise. The optimized variant is then capable of automatically denoising the input data and thus generating more robust feature representations compared to a basic autoencoder for the current recognition tasks.

#### 3.2.3. An Improved Shared-Hidden-Layer Autoencoder (SHLA)

**Figure 3:**
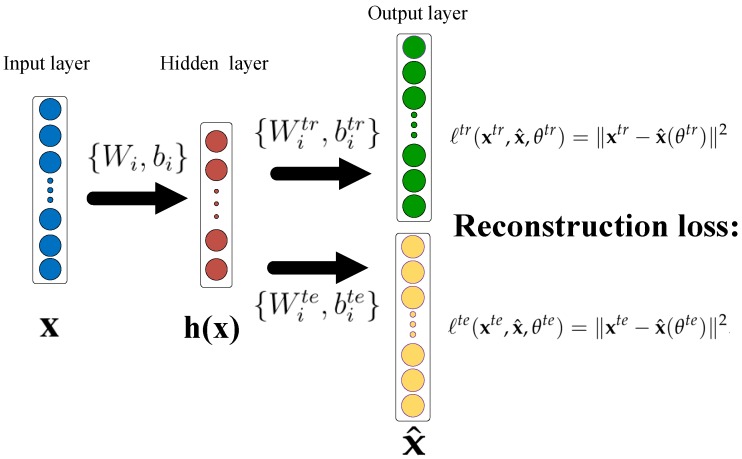
The architecture of the Shared-Hidden-Layer Autoencoder (SHLA) model.

Similar with idea of the transfer learning, another effective variant of the basic autoencoder, named SHLA [48], was just based on the motivation of the ’sharing idea’. The idea behind SHLA is that the autoencoder shares the same parameters for the mapping from the input layer to the hidden layer, but adopts the independent parameters for the reconstruction process. SHLA was proposed to minimize the reconstruction error on both training set and test set, which is shown in Figure 3.

Given the training dataset $x^{tr}$, and the test dataset $x^{te}$, then the two loss functions are formulated as:

(5) $$\ell^{tr}\left(x^{tr},\hat{x},\theta^{tr}\right)={∥x^{tr}-\hat{x}\left(\theta^{tr}\right)∥}^{2}.$$

(6) $$\ell^{te}\left(x^{te},\hat{x},\theta^{te}\right)={∥x^{te}-\hat{x}\left(\theta^{te}\right)∥}^{2}.$$

where $\theta^{tr}=\left\{W_{i},b_{i},W_{i}^{tr},b_{i}^{tr}\right\}$ and $\theta^{te}=\left\{W_{i},b_{i},W_{i}^{te},b_{i}^{te}\right\}$ are the parameters of the training period and testing period, respectively. It can be seen from the expression above, the two functions share the same parameters $\left\{W_{i},b_{i}\right\}$, which represent the connections from the input to the hidden layer.

Moreover, in [48], to optimize the joined distance for the two sets, the following loss function was formed as:

(7) $$\ell^{all}\left(\theta^{all}\right)=\ell^{tr}\left(x^{tr},\hat{x},\theta^{tr}\right)+\lambda \ell^{te}\left(x^{te},\hat{x},\theta^{te}\right).$$

Finally, the following overall objective function was defined as:

(8) $$L\left(\theta^{all}\right)=\min_{\theta^{all}}\ell^{all}\left(\theta^{all}\right)+\gamma_{1}(∥W^{tr}{∥}_{1}+{∥W∥}_{1})+\gamma_{2}{∥W^{te}∥}_{1}.$$

where the parameters $\theta^{all}=\left\{W_{i},b_{i},W_{i}^{tr},b_{i}^{tr},W_{i}^{te},b_{i}^{te}\right\}$ are optimized in the period of training. The λ is the hyper-parameter which acts as a weight-decay regularization term, and the hyper-parameters $\left\{\gamma_{1},\gamma_{2}\right\}$ control the strength of the regularization.

Based on the basic SHLA model, an improved SHLA model is proposed to generate high-level feature representations from the hidden layers in our work. There are two differences between the basic and improved SHLA model. The first one lies in the final objective function in Equation (8) where we change the regularization term, and the second is that the improved SHLA model is followed with an auxiliary layer to jointly exploit useful intrinsic associations from multiple original features.

**Figure 4:**
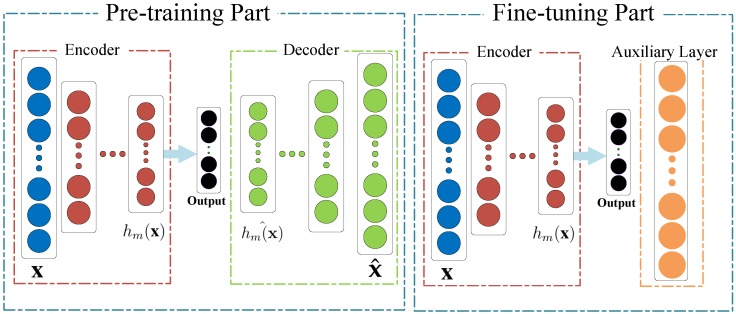
The architecture of each branch network in heterogeneous unification module: The pre-training part (**left**) and the fine-tuning (**right**).

From Figure 1, the heterogeneous unification module is composed of several branch networks which correspond to different kinds of features. In addition, we can see from Figure 4 that each branch network of heterogeneous unification module contains two parts of different periods: Pre-training part and fine-tuning part.

In the period of the unsupervised pre-training, the branch network, which is made up of multiple hidden layers, is pre-trained layer-wisely by feeding the various low-level heterogeneous features. The output of the previous hidden layer from the encoder acts as the input to the subsequent hidden layer through minimizing the reconstructing error by subtracting the reconstructed data from the original input data. As for the different kinds of features, the corresponding branch network is different from each other. In other words, the architecture of each branch network, including the structure of hidden layers and number of the hidden nodes, is different.

Then, in the supervised fine-tuning process, the decoder is replaced with a auxiliary layer which is shared by all the branch networks, as shown in Figure 4. The auxiliary layer, which includes supervised information such as classification results or labels, is utilized for fine-tuning the whole branch networks. In addition, the main idea of the fine-tuning part is to exploit the intrinsic associations among those multiple heterogenous features.

Finally, the original heterogeneous features, which are processed by the proposed architecture during the pre-training stage and fine-tuning stage in turn, are converted into the unified representations. Likewise, stochastic gradient descent acts as the strategy in the training of the heterogeneous unification module.

In the following experiments, these three types of models, basic autoencoders, denoising autoencoders and the improved SHLA, are employed to investigate the efficacy of the proposed architecture through comparing the classification performance.

### 3.3. Fusion Network Module

A simple fusion strategy is utilized to enhance the performance of speech emotion recognition task because of the power of unified feature representation obtained from heterogeneous unification module. The fusion network module in this paper is a four-layers deep neural network which contains one input layer and three hidden layers.

As can be seen from the Figure 1, for instance, five refined and unified high-level abstract features generated from the branch networks in the heterogeneous unification module are concatenated to form a joint feature representation. The fusion network module is utilized to capture the associations between those unified joint features for emotion recognition task by taking full advantage of DNNs. As a result, a 1024-dimensional feature vector is acquired from the last hidden layer which acts as the final acoustic feature representation.

Additionally, to evaluate the performance of several classifiers, an SVM is utilized as the final classifier to perform the prediction in our achitecture.

## 4. Experiment

### 4.1. Dataset

We evaluate our proposed architecture on the dataset named Interactive Emotional Dyadic Motion Capture Database (IEMOCAP) [31] collected at Signal Analysis and Interpretation Laboratory (SAIL) at university of Southern California (USC). The IEMOCAP dataset is an acted, multimodal emotion corpus including audio, visual and text data which was recorded from ten actors during dyadic interactions. The dataset is organized in five sessions and two actors are involved in improvisations or scripted scenarios designed to elicit specific emotional expressions. The corpus contains approximately 12 h of recordings with detailed motion capture information carefully synchronized with audio. Sessions are manually segmented into utterances. Each sentence in this dataset was assigned one emotion label by at least 3 human annotators, such as happy, sad, neutral, anger, surprised, excited, disgust, fear, and so on. In this paper, we only utilize the audio data for the experiment. Followed the design of the previous work [50], we use audio signals from four emotional categories of angry (1103), happy (1636), neutral (1708) and sad (1084). The category distribution and duration of IEMOCAP dataset are given in Table 1.

### 4.2. Result Analysis and Discussion

To testify the effectiveness of the proposed framework for emotion recognition, we conducted several experiments on the IEMOCAP dataset. In this work, per-class emotion accuracy and total accuracy are adopted for comparison and analysis according to different methods.

It is well known that selecting an appropriate classifier is very important for a recognition system. In order to find out the best efficient classifier first, we evaluate the proposed architecture by using different classifiers including K-Nearest Neighbor (KNN), LR, Random Forest (RF) and SVM.

As for those classifiers, how to select hyper parameters is one of the key factors in the success of a classification model. In this work, these hyper parameters are determined according to the accuracy obtained on the validation set. For SVM classifier, we tested several types of kernel functions, such as linear kernel, RBF, Poly, ect., to find an appropriate kernel function. Furthermore, we performed n-fold cross validation strategy to find the optimal parameters *c* in the range of [0.001, 10] and σ in the range of [0.001, 8]. And for RF classifier, the number of trees is selected from 50 to 900 with 50 step length and the depth of the tree is searched from 2 to 18.

In comparison with these three different classifiers in Table 2, SVM classifier achieved the best performance for emotion recognition task than other classification techniques. The classification accuracy achieved by SVM is 9% higher than KNN classifier which is the maximum gap between the results of the four classifiers. Observed from the table, SVM classifier and RF classifier have achieved similar results in classification performance on IEMOCAP dataset of four categories. As a result, because of its superior classification performance on IEMOCAP database in this experiment, the SVM is adopted as the final classifier in this study.

As shown in Table 2, the SVM classifier has best performance with 79% accuracy on **Happy** category and the worst performance with 45% accuracy on **Neutral** category. Actually, since **Neutral** is the neutral state of a person, so **Neutral** category could be easily confused with other emotional states, leading to the worst classification result. Compared to the **Happy** category, the classification results of **Angry** and **Sad** are not good because the two categories have relatively small number of samples. From Table 2, we observe that the similar phenomenon happens when using other classifiers.

In addition, another experiment is adopted to exploit the efficiency of the different single acoustic feature, including IS10, MFCCs, eGemaps, SoundNet bottleneck feature and VGGish bottleneck feature which are processed by heterogeneous unification module. Table 3 illustrates the comparison of the classification results of different features where SVM acts as the classifier to do the prediction.

From experimental results in Table 3, we find that different acoustic features have the different classification abilities for current task. Among those acoustic features, deep learning based bottleneck features significantly outperform the low-level features which were widely used and effective in previous works. As can be seen from the table, the biggest gap of classification accuracy is 13 percent compared to the results by using the VGGish bottleneck feature and MFCCs feature set, respectively. Obviously, the results in this experiment show that deep neural models have exhibited mighty feature learning ability.

As stated from Table 3, we can find that the best recognition performance is achieved by using VGGish bottleneck feature. Benefiting by the delicately selected network from various CNN architectures and superior efficiency of bottleneck features extracted from well fine-tuned model [46], VGGish bottleneck feature achieves the highest performance with 48% accuracy compared with other single feature set in this work.

Furthermore, to benchmark the proposed method, we conducted the experiments compared with the existing approaches in previous research on the IEMOCAP dataset. We carry out comparative experiments between the two representative existing techniques, including Lakomkin et al. [29] and Gu et al. [30].

From Table 4, we observe that Lakomkin et al. [29] achieved the performance with 58% accuracy and Gu et al. [30] achieved the performance with 62% accuracy on the IEMOCAP dataset. Compared with state-of-the-art results mentioned above, our experimental results indicate that the proposed method named ours + shla where the improved SHLA model acts as the branch network in the heterogeneous module, achieves the better classification performance than those approaches in previous researches for speech emotion recognition. For example, in Lakomkin et al. [29], two models which use a pre-trained automatic speech recognition (ASR) network were proposed for speech emotion recognition. They just considered using various neural architectures to generate speech features, however we make full use of low-level and high-level features for classification.

The results shown in Table 4 demonstrate that our architecture could learn discriminative information from multiple heterogeneous features and achieve the competitive classification performance in speech emotion recognition task.

Specifically, to investigate the effect of each module of the proposed architecture, we perform a series of ablation studies in this work. Per-class emotion accuracy comparison of ablation studies is illustrated in Table 5.

In Table 5, the method named ours-H denotes that the heterogeneous unification module is removed from the proposed architecture. To be specific, we just simply concatenate multiple features from features extraction module into a high-dimensional feature vector, which acts as the input of fusion network module. The second method named ours-F in Table 5 denotes that the fusion network module is removed from the proposed architecture. The third method named ours + dae denotes that DAE model acts as the branch network in the heterogeneous module. The last method named ours + shla denotes that the improved SHLA model acts as the branch network.

The ours-H method obtains the performance with 51% accuracy which is the worst result in the experiments. There is a wide gap between this result and the best one (64%) achieved by the proposed method named ours + shla. This further verifies that the heterogenous unification module is exhibiting great superiority in the whole architecture.

The ours-F method reaches 59% accuracy which demonstrates that the fusion network module also can increase the classification accuracy by 5% compared to ours + shla method. This experimental result proves that the fusion network module designed in this work is very useful for achieving good classification results.

We further conducted a experiment to evaluate the different models used in branch network of the heterogeneous module. In Table 5, ours + dae method by using DAE as the branch network in the heterogeneous module obtains accuracy of 63%. The results demonstrate that slight improvement is acquired by using the improved SHLA model compared to DAE model.

From these above mentioned studies, two important conclusions can be drawn: (1) The heterogenous unification module could unify the heterogeneous representation to improve performance and (2) the fusion network module is useful for better performance in this work.

## 5. Conclusions and Future Work

In this paper, we proposed a speech emotion recognition architecture that solved the acoustic features heterogeneous problem which generally deteriorates the classification performance. The proposed hybrid deep neural network mainly consists of a features extraction module, a heterogeneous unification module and a fusion network module. Instead of the multiple heterogeneous features, the refined and unified features are fed into the fusion network module for current recognition task. Experimental results performed on the IEMOCAP dataset showed that the proposed architecture can work effectively and achieve competitive classification performance compared to several baseline approaches. The proposed deep neural network and the approaches in this paper can also be applied in the other research areas to use the best of the multiple and heterogeneous features for better classification performance. In the future, we will try to do more experiments on other public benchmark databases to analyze our work. The other direction of research is to use this architecture to deal with multimodal features for emotion recognition.

## Figures and Tables

**Table 1:** Different emotion category distribution and duration (interactive emotional dyadic motion capture database—IEMOCAP).

Emotion	Angry	Happy	Neutral	Sad	Total
Utterances	1103	1636	1708	1084	5531
Duration (min)	83.0	126.0	111.1	99.3	419.4

**Table 2:** Comparison of the classification results of different classifiers.

Classifiers	KNN	LR	RF	SVM
**Angry**	0.56	0.64	0.66	0.65
**Happy**	0.71	0.73	0.77	0.79
**Neutral**	0.38	0.39	0.46	0.45
**Sad**	0.58	0.62	0.64	0.69
**Total**	0.55	0.59	0.63	0.64

**Table 3:** Per-class Emotion Accuracy Comparison of the different features.

Features	IS10	MFCCs	eGemaps	SoundNet	VGGish
**Angry**	0.39	0.33	0.43	0.47	0.49
**Happy**	0.53	0.51	0.57	0.59	0.63
**Neutral**	0.21	0.21	0.24	0.29	0.3
**Sad**	0.42	0.37	0.43	0.48	0.51
**Total**	0.38	0.35	0.41	0.45	0.48

**Table 4:** Per-class emotion accuracy comparison of different approaches.

Approaches	Angry	Happy	Neutral	Sad	Total
Lakomkin [29]	0.59	0.72	0.37	0.59	0.58
Gu [30]	-	-	-	-	0.62
**ours + shla**	0.65	0.79	0.45	0.69	0.64

**Table 5:** Per-class emotion accuracy comparison of ablation studies.

Methods	Angry	Happy	Neutral	Sad	Total
**ours-H**	0.53	0.64	0.33	0.56	0.51
**ours-F**	0.61	0.74	0.41	0.62	0.59
**ours + dae**	0.63	0.79	0.45	0.66	0.63
**ours + shla**	0.65	0.79	0.45	0.69	0.64
